# Ferroptosis, necroptosis, pyroptosis, and cuproptosis in cancer: a comparative bibliometric analysis

**DOI:** 10.1038/s41420-023-01542-7

**Published:** 2023-07-10

**Authors:** Haiyang Wu, Kunming Cheng, Cheng Li

**Affiliations:** 1 Department of Clinical College of Neurology, Neurosurgery and Neurorehabilitation, Tianjin Medical University, Tianjin, China; 2 Duke Molecular Physiology Institute, Duke University School of Medicine, Durham, North Carolina USA; 3 Department of Intensive Care Unit, The Second Affiliated Hospital of Zhengzhou University, Zhengzhou, China; 4 Department of Orthopaedic Surgery, Beijing Jishuitan Hospital, Fourth Clinical College of Peking University, Beijing, China; 5 State Key Laboratory of Toxicology and Medical Countermeasures, Beijing Institute of Pharmacology and Toxicology, Beijing, China; 6 Center for Musculoskeletal Surgery (CMSC), Charité-Universitätsmedizin Berlin, corporate member of Freie Universität Berlin, Humboldt University of Berlin, and Berlin Institute of Health, Berlin, Germany

**Keywords:** Cell death, Cancer

We recently read the publication by Miao et al., “A bibliometric analysis of ferroptosis, necroptosis, pyroptosis, and cuproptosis in cancer from 2012 to 2022” [1] with great interest. This study aimed to elucidate the knowledge structures, development trends and research hotspots of ferroptosis, necroptosis, pyroptosis, and cuproptosis in cancer. Given the importance of these cell death modalities in cancer, the study’s significance merits recognition [2, 3]. However, we have some suggestions regarding the retrieval strategies employed in this study.

For bibliometric study, the search strategy is very important. The authors mentioned that the original data were acquired from Web of Science Core Collection (WoSCC). To our knowledge, WoSCC comprises several sub-databases, including SCI-Expanded, SSCI, A&HCI, CPCI-S, CPCI-SSH, and others. Previous studies and our experience suggest that not all sub-databases are appropriate for bibliometric analysis [4]. Among them, SCI-Expanded is the most appropriate and widely used one. Therefore, the author should clearly specify which database they utilized for data repeatability. Additionally, several researchers argue that the Topic Search (TS) is not suitable for bibliometric analysis. TS considers a study to be the target study when search terms appear in “Title (TI)”, “Abstract (AB)”, “Author keywords (AK)”, or “Keywords plus”. However, “Keywords plus” is generated by an automatic computer algorithm of WoSCC, not from the authors. Including “Keywords Plus” during the search process may result in the inclusion of many unrelated publications [4, 5]. In our experience, using “TI”, “AB”, and “AK” as the qualification maybe the best option.

Moreover, if the search formula is overly simplistic and omits related keywords, it may overlook many relevant publications. For example, in this study, the author used only “cancer” and “tumor” to identify cancer-related literature. We contend that this is insufficient for locating all cancer-related studies. Our previous work offers a detailed example of searching for cancer-related studies using the following terms: “cancer* OR anticancer* OR tumor* OR tumour* OR oncology OR neoplasm* OR carcinoma* OR lymphoma* OR sarcoma* OR leukemia*” [6]. In addition, the authors could improve the search terms by incorporating wildcard characters such as “*”. The wildcard character “*” can replace any other characters, allowing for variable keyword endings. For example, “cancer*” would return both “cancer” and “cancers”. Furthermore, we recommend the authors use “ferroptosis OR ferroptotic” instead of “ferroptosis”, “necroptosis OR necroptotic” instead of “necroptosis”, “pyroptosis OR pyroptotic OR inflammasome OR pyroptosome” instead of “pyroptosis”, “cuproptosis OR ((copper-induced OR copper-mediated) AND (cell death*))” instead of “cuproptosis”. Our suggested retrieval formula is summarized in additional file 1.

Based on the updated search method, from January 1, 2012, to December 31, 2022, we retrieved 3363 (ferroptosis in cancer), 1998 (necroptosis in cancer), 3672 (pyroptosis in cancer), and 246 (cuproptosis in cancer) records. The annual publication trend in the four areas is shown in Fig. 1. Compared to the results from Miao et al., although the retrieval scope in our study narrows down to TI/AK/AB, we identified more related studies in three of areas, particularly pyroptosis in cancer (3672 vs 1445). Of note, as the number of publications has significantly changed, many quantitative data points, such as the most prolific countries, institutions, and authors, will also be affected. Therefore, in order to avoid this kind of bias, determining the appropriate retrieval formula is a crucial step for bibliometric analysis. In our opinion, seeking expert consultation on search keywords within the specific field is essential.

**Fig. 1:**
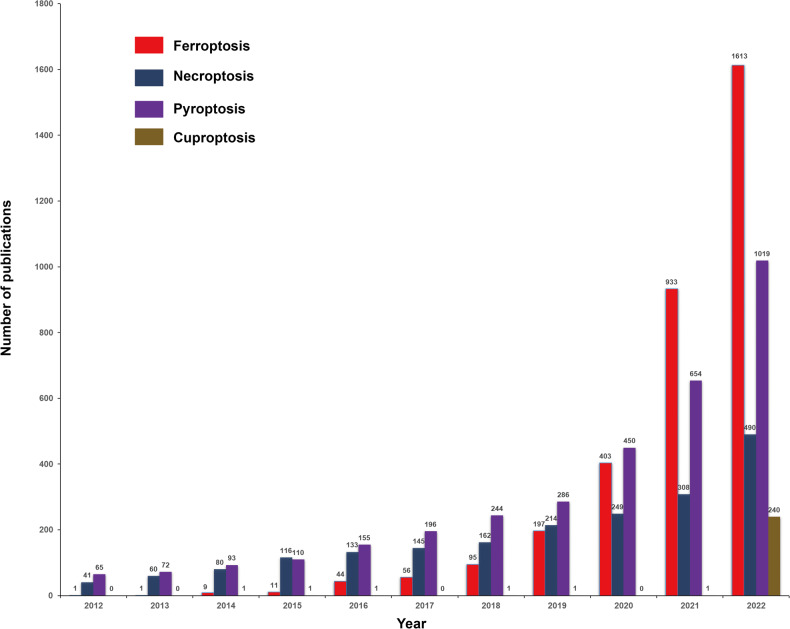
The annual publication trend in the four areas.

Lastly, the authors claimed that this study was the first bibliometric analysis of “ferroptosis in cancer”, “necroptosis in cancer”, “pyroptosis in cancer”, and “cuproptosis in cancer” using VOSviewer software. However, several works have already conducted bibliometric analyses of ferroptosis in cancer [7, 8]. In conclusion, we congratulate Miao et al. for their work, but we believe that our suggestions could provide more accurate data to analyze the research trends of ferroptosis, necroptosis, pyroptosis, and cuproptosis in cancer.

## Supplementary information

our suggested retrieval formula
