# Supplementary material for: Ferroptosis, necroptosis, pyroptosis, and cuproptosis in cancer: a comparative bibliometric analysis
Source: Cell Death Discov. 2023 Jul 10;9:238. doi: 10.1038/s41420-023-01542-7 (PMC10333212; doi:10.1038/s41420-023-01542-7)
Supplement: Supplementary file 1 — our suggested retrieval formula [file 41420_2023_1542_MOESM1_ESM.docx]

Ferroptosis:

(TI=(cancer* OR anticancer* OR tumor* OR tumour* OR oncology OR neoplasm* OR carcinoma* OR lymphoma* OR sarcoma* OR leukemia*) OR AK=(cancer* OR anticancer* OR tumor* OR tumour* OR oncology OR neoplasm* OR carcinoma* OR lymphoma* OR sarcoma* OR leukemia*) OR AB=(cancer* OR anticancer* OR tumor* OR tumour* OR oncology OR neoplasm* OR carcinoma* OR lymphoma* OR sarcoma* OR leukemia*)) AND (TI=(ferroptosis OR ferroptotic) OR AK=(ferroptosis OR ferroptotic) OR AB=(ferroptosis OR ferroptotic))

Necroptosis

(TI=(cancer* OR anticancer* OR tumor* OR tumour* OR oncology OR neoplasm* OR carcinoma* OR lymphoma* OR sarcoma* OR leukemia*) OR AK=(cancer* OR anticancer* OR tumor* OR tumour* OR oncology OR neoplasm* OR carcinoma* OR lymphoma* OR sarcoma* OR leukemia*) OR AB=(cancer* OR anticancer* OR tumor* OR tumour* OR oncology OR neoplasm* OR carcinoma* OR lymphoma* OR sarcoma* OR leukemia*)) AND (TI=(necroptosis OR necroptotic) OR AK=(necroptosis OR necroptotic) OR AB=(necroptosis OR necroptotic))

Pyroptosis

(TI=(cancer* OR anticancer* OR tumor* OR tumour* OR oncology OR neoplasm* OR carcinoma* OR lymphoma* OR sarcoma* OR leukemia*) OR AK=(cancer* OR anticancer* OR tumor* OR tumour* OR oncology OR neoplasm* OR carcinoma* OR lymphoma* OR sarcoma* OR leukemia*) OR AB=(cancer* OR anticancer* OR tumor* OR tumour* OR oncology OR neoplasm* OR carcinoma* OR lymphoma* OR sarcoma* OR leukemia*)) AND (TI=(pyroptosis OR pyroptotic OR inflammasome OR pyroptosome) OR AK=(pyroptosis OR pyroptotic OR inflammasome OR pyroptosome) OR AB=(pyroptosis OR pyroptotic OR inflammasome OR pyroptosome))

Cuproptosis

(TI=(cancer* OR anticancer* OR tumor* OR tumour* OR oncology OR neoplasm* OR carcinoma* OR lymphoma* OR sarcoma* OR leukemia*) OR AK=(cancer* OR anticancer* OR tumor* OR tumour* OR oncology OR neoplasm* OR carcinoma* OR lymphoma* OR sarcoma* OR leukemia*) OR AB=(cancer* OR anticancer* OR tumor* OR tumour* OR oncology OR neoplasm* OR carcinoma* OR lymphoma* OR sarcoma* OR leukemia*)) AND (TI=(cuproptosis OR ((“copper-induced” OR “copper-mediated”) AND (cell death*))) OR AK=(cuproptosis OR ((“copper-induced” OR “copper-mediated”) AND (cell death*))) OR AB=(cuproptosis OR ((“copper-induced” OR “copper-mediated”) AND (cell death*))))
